# Supplementary figures and images for: Identification of potential human pancreatic α-amylase inhibitors from natural products by molecular docking, MM/GBSA calculations, MD simulations, and ADMET analysis
Source: PLoS One. 2023 Mar 16;18(3):e0275765. doi: 10.1371/journal.pone.0275765 (PMC10019617; doi:10.1371/journal.pone.0275765)

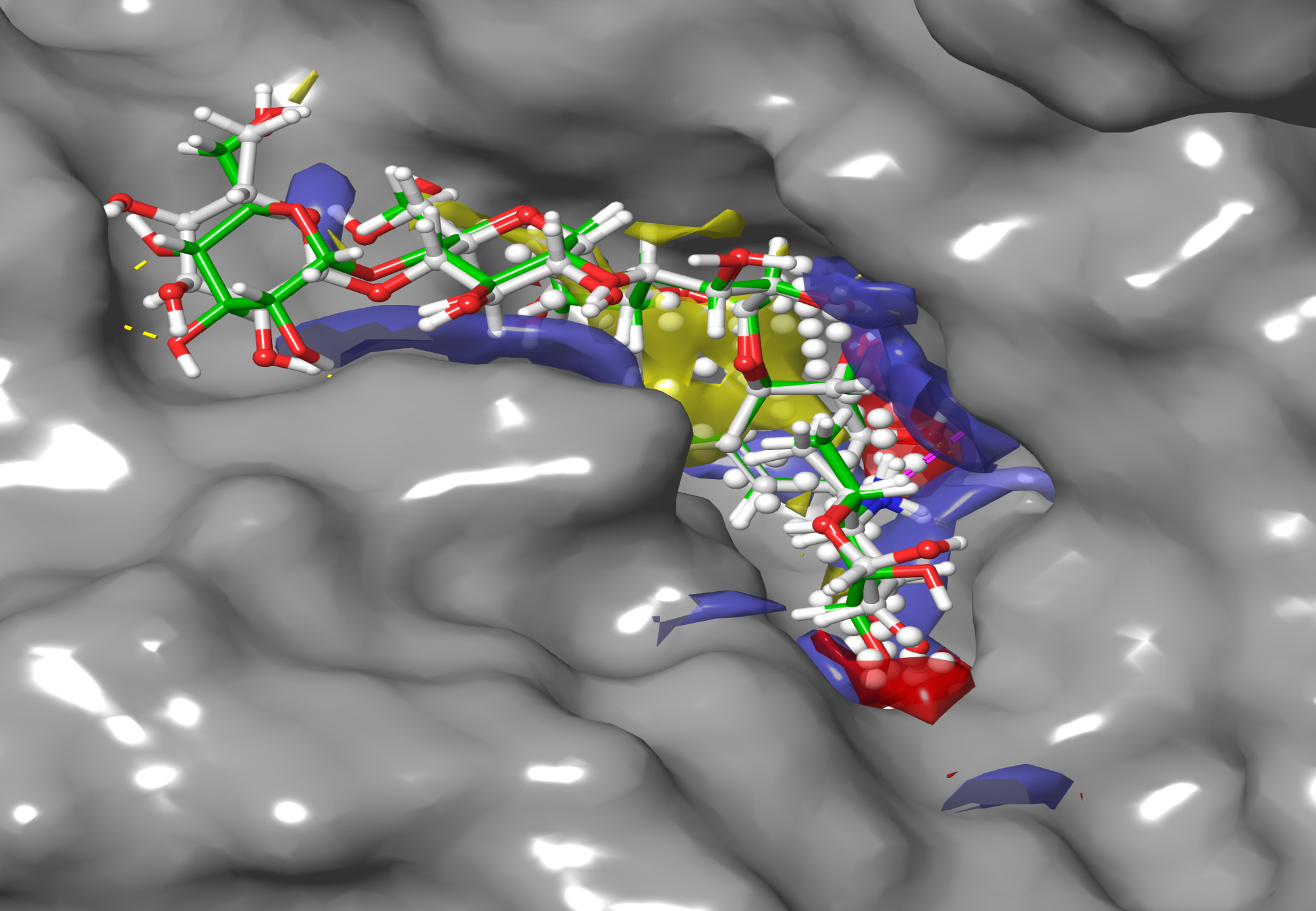

Supplement: S1 Fig — The yellow and blue patches represent the SiteMap application view. (TIF) [file pone.0275765.s001.tif]

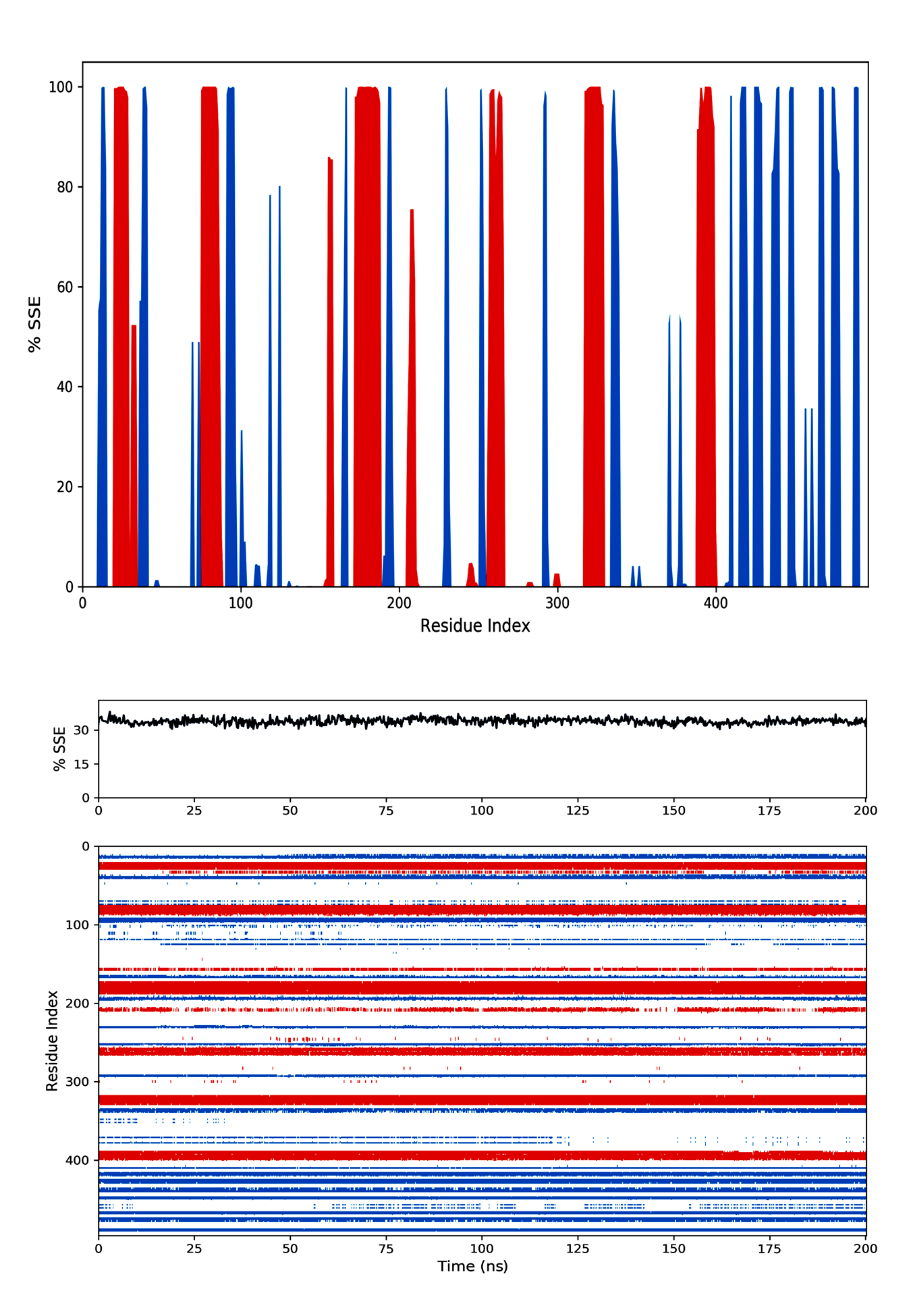

Supplement: S2 Fig — (TIF) [file pone.0275765.s002.tif]

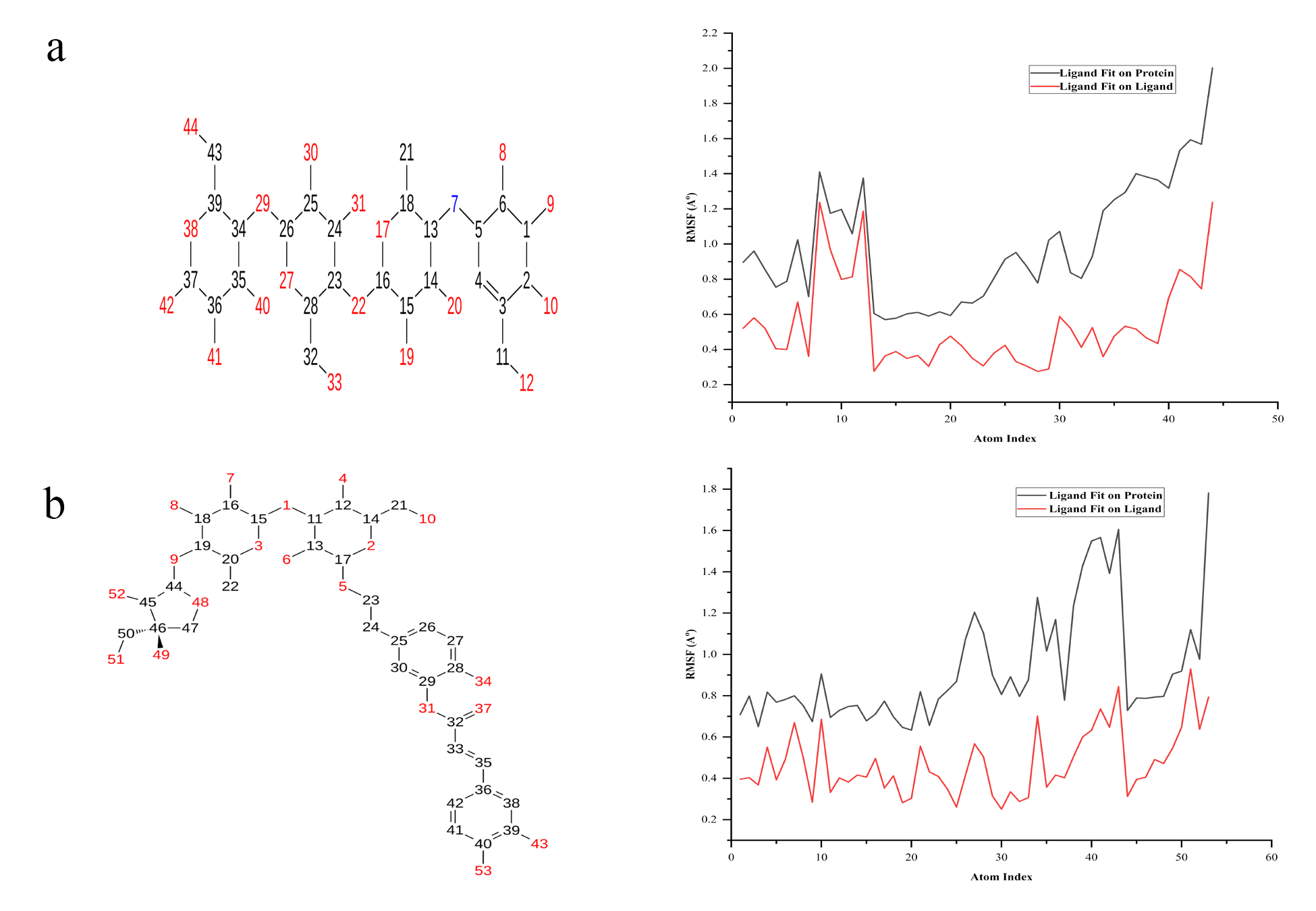

Supplement: S3 Fig — (TIF) [file pone.0275765.s003.tif]

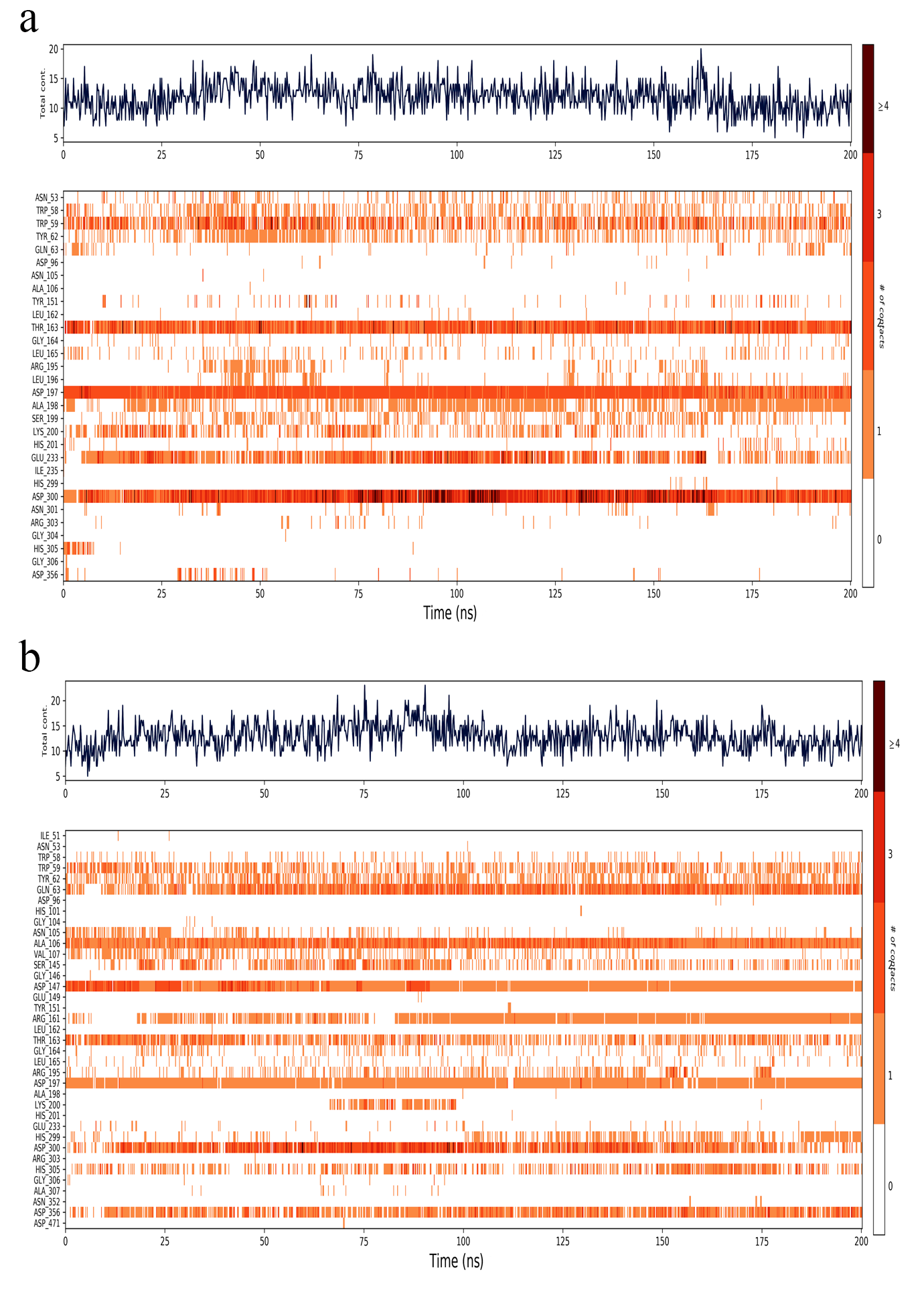

Supplement: S4 Fig — (TIF) [file pone.0275765.s004.tif]

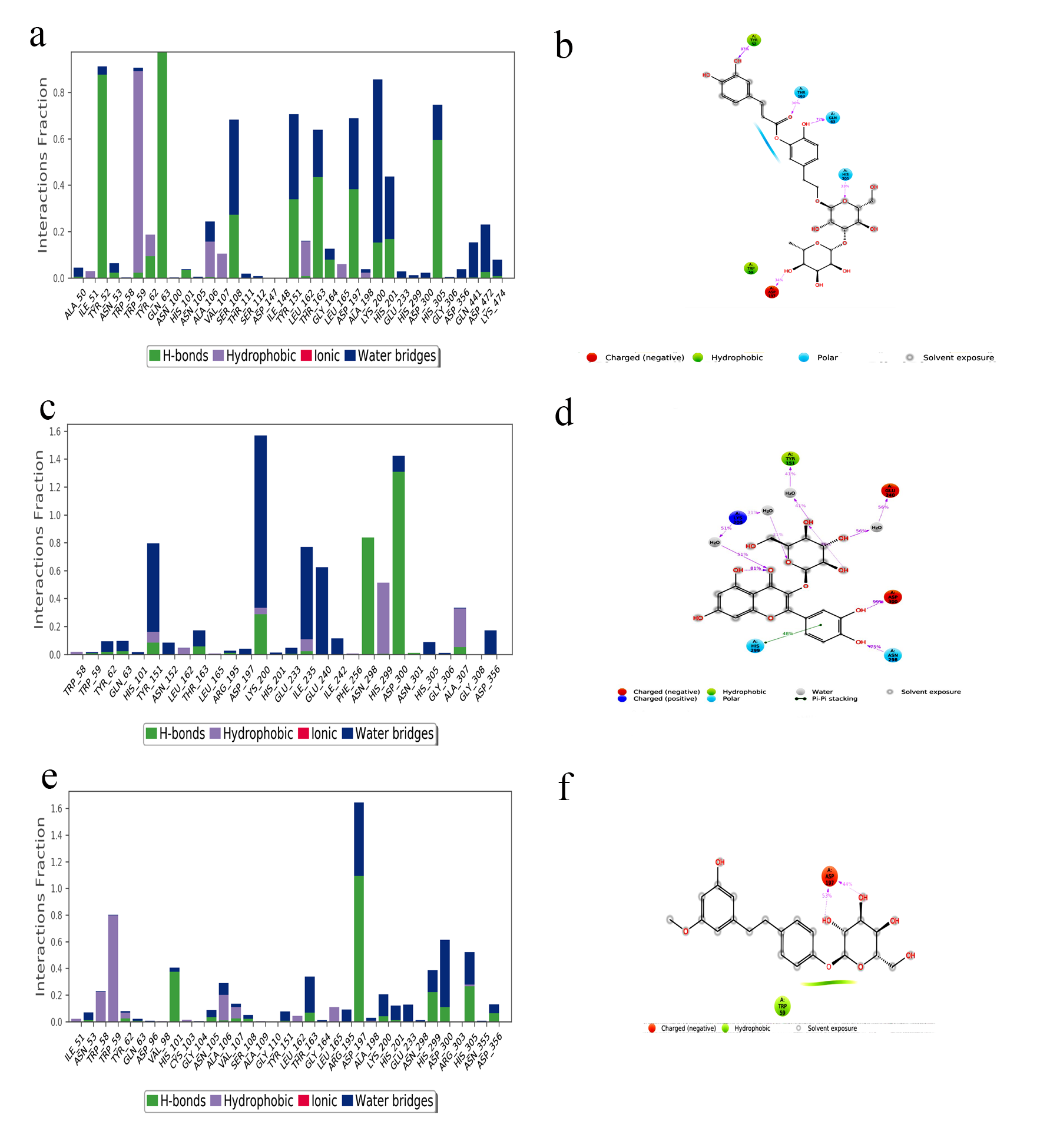

Supplement: S5 Fig — Analysis of inhibitor’s molecular interaction and type of contact with HPA after MD simulations: Normalized stacked bar chart of HPA binding site residues interacting with (a) newboulaside A, (c) quercetin-3-O-β-glucoside, and (e) sasastilboside A via hydrogen bonds, hydrophobic and ionic interactions and water bridges. Detailed schematic interaction of (b) newboulaside A, (d) quercetin-3-O-β-glucoside, and (f) sasastilboside A atoms with the binding site residues of HPA. Interactions happening more than 30% of the simulation times are shown. (TIF) [file pone.0275765.s005.tif]

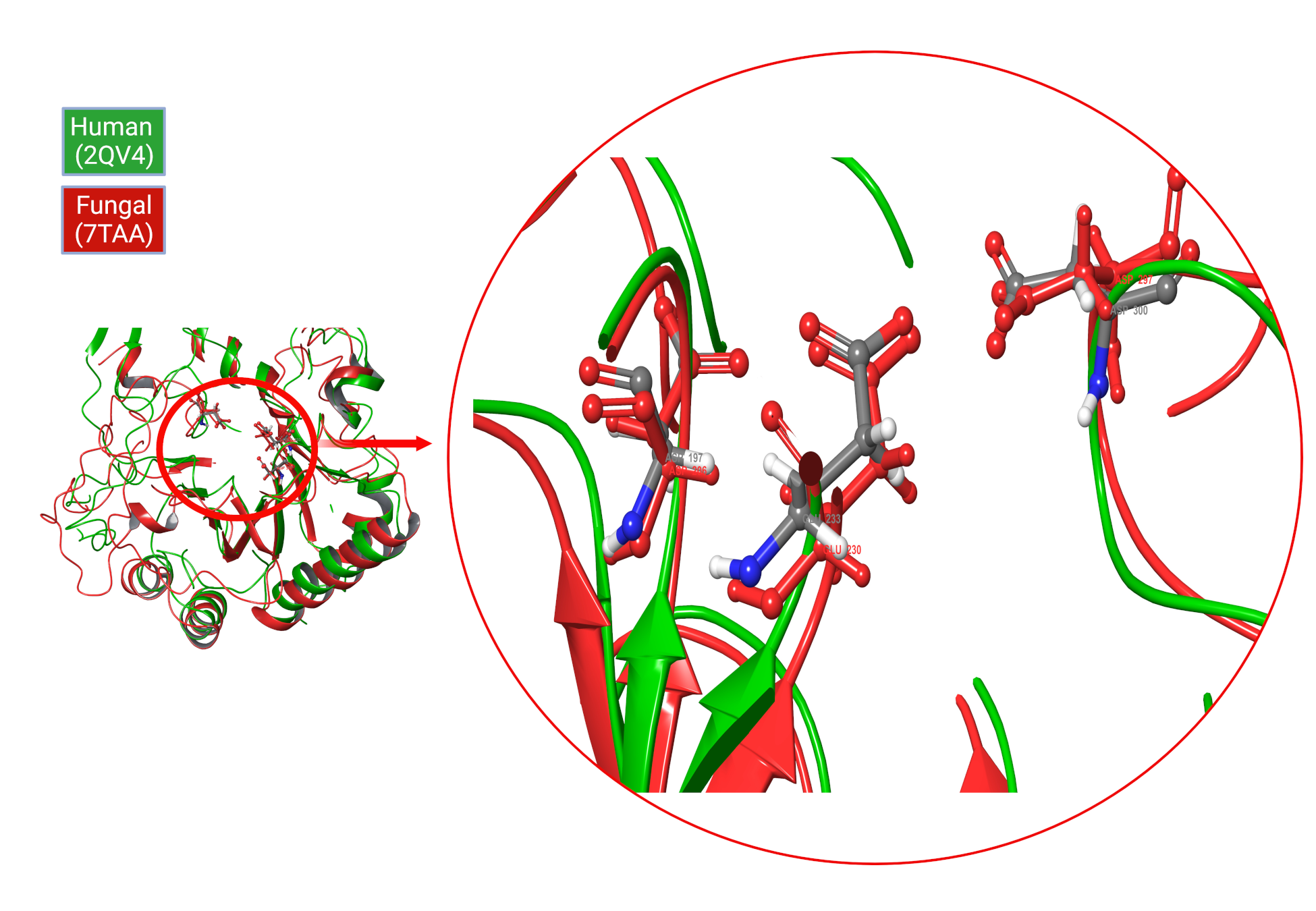

Supplement: S6 Fig — (TIF) [file pone.0275765.s006.tif]

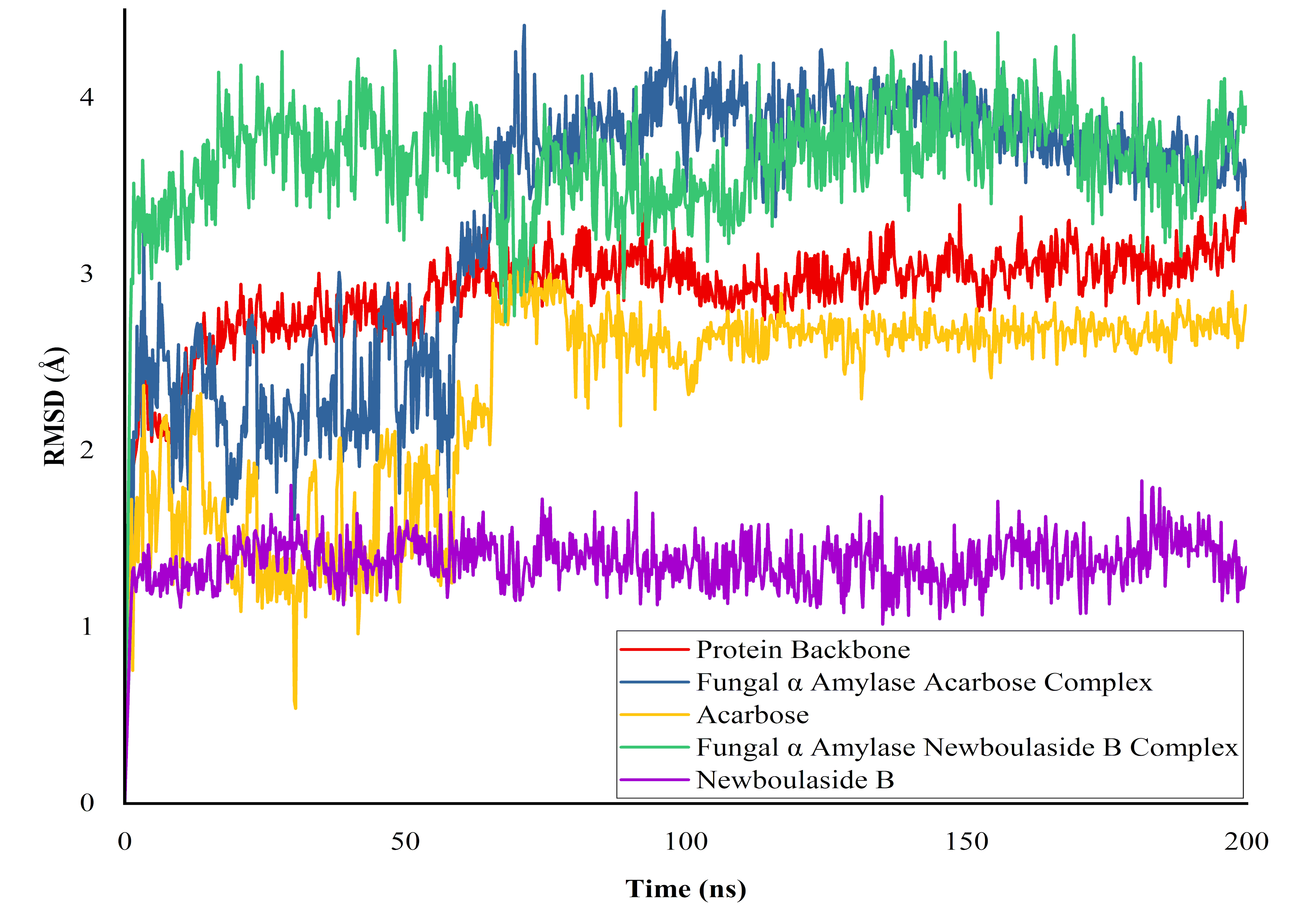

Supplement: S7 Fig — (TIF) [file pone.0275765.s007.tif]
