# Supplementary material for: Identification of potential human pancreatic α-amylase inhibitors from natural products by molecular docking, MM/GBSA calculations, MD simulations, and ADMET analysis
Source: PLoS One. 2023 Mar 16;18(3):e0275765. doi: 10.1371/journal.pone.0275765 (PMC10019617; doi:10.1371/journal.pone.0275765)
Supplement: S1 Table — (DOCX) [file pone.0275765.s008.docx]

**Supplementary Material**

**Identification of potential human pancreatic *α*-amylase inhibitors from natural products by molecular docking, MM/GBSA calculations, MD simulations, and ADMET analysis**

Santosh Basnet^1^**^¶^**, Madhav Prasad Ghimire^2&^, Tika Ram Lamichhane^2&^, Rajendra Adhikari^3&^, Achyut Adhikari^1&*^

^1^ Central Department of Chemistry, Tribhuvan University, Kirtipur, Kathmandu, Nepal

^2^ Central Department of Physics, Tribhuvan University, Kirtipur, Kathmandu, Nepal

^3^ Department of Physics, Kathmandu University, Dhulikhel, Nepal

^*^ Corresponding author: [achyutraj05@gmail.com](mailto:achyutraj05@gmail.com)

Table S1. Ligand library of natural products

| Source of  Inhibitor | Inhibitor | Structure | IC_50_ /% of  Inhibition | Ref |
| --- | --- | --- | --- | --- |
| *Ocimum tenuiflorum* | dehydrodieugenol B |  | 29.6 μM | [1] |
| Olive mill  wastes (OMW) | 1-acetoxypinoresinol |  | 13.9 μM | [2] |
| *Sargassum patens* | DDBT |  | 3.2μg/mL | [3] |
| *Humulus lupulus* | 3ʹ-geranyl  chalconaringenin |  | 20.46 μM | [4] |
| *Azadirachta indica* | gedunin |  | 68.38 μM | [5] |
| *Dioscorea bulbifera* | diosgenin |  | 70.94 % | [6] |
| *Swertia chirata* | mangiferin |  | 516.66 μM | [7] |
| *Syzygium cumini* | ursolic acid |  | 6.7 μg/mL | [8] |
| *Syzygium cumini* | oleanolic acid |  | 57.4  μg/mL | [8] |
| *Abrus precatorius* | lupenone |  | 31 μM | [9] |
| *Psidium guajava Linn* | myricetin |  | 4.3 mM | [10] |
| *Punica granatum* | valoneic acid dilactone |  | 0.284  μg/mL | [11] |
|  | acarbose |  | Standard |  |
| *Passiflora ligularis* Juss | quercetin-3-O-*β*-glucoside |  | 31.0μM | [12] |
| *Passiflora ligularis* Juss | kaempferol-3-O-*β*-glucoside |  | 33.4μM | [12] |
| *Passiflora ligularis* Juss | ligularoside A |  | 409.8 μM | [12] |
| *Rheum turkestanicum* | daucosterol |  | 46.4 μM | [13] |
| *Rheum turkestanicum* | rhododendrin |  | 107.5μM | [13] |
| *Rheum turkestanicum* | emodin |  | 184.7 μM | [13] |
| *Zanthoxylum chalybeum* | chaylbemide A |  | 45.76 μM | [14] |
| *Zanthoxylum chalybeum* | chaylbemide B |  | 43.22 μM | [14] |
| *Zanthoxylum chalybeum* | chaylbemide C |  | 46.76 μM | [14] |
| *Zanthoxylum chalybeum* | trans-fagaramide |  | 47.36 μM | [14] |
| *Zanthoxylum chalybeum* | skimmianine |  | 47.72 μM | [14] |
| *Zanthoxylum chalybeum* | norchelerythrine |  | 46.49 μM | [14] |
| *Zanthoxylum chalybeum* | sesamine |  | 54.67 μM | [14] |
| *Musa acuminate* | cycloeucalenone |  | 20.33 μM | [15] |
| *Dipterocarpus littoralis* | *α*-viniferin |  | 212.79 μg/mL | [16] |
| *Melicope latifolia* | halfordin |  | 197.53 μM | [17] |
| *Melicope latifolia* | *β*-sitosterol |  | 372.31 μM | [17] |
| *Newbouldia laevis* | newboulaside A | 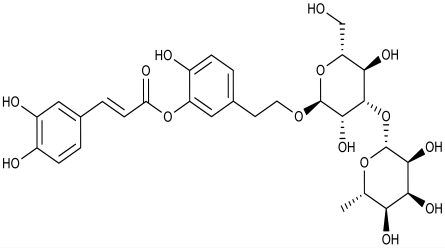 | 4.95 µg/mL | [18] |
| *Newbouldia laevis* | newboulaside B | 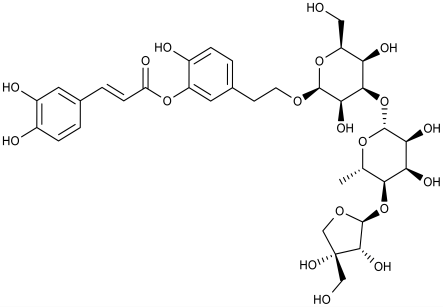 | 4.44 µg/mL | [18] |
| *Camellia sasanqua* | sasastilboside A |  | 53.7 μM | [19] |
| *Salvia virgata* | chrysoeriol |  | 1.27 mM | [20] |
| *Oryza sativa* cv | momilactone A |  | 266.68 μg/mL | [21] |
| *Oryza sativa* cv | momilactone B |  | 146.85  μg/mL | [21] |
| *Setosphaeria rostrata* | rostratazine B |  | 578 μM | [22] |
| *Physalis peruviana* | peruviose D |  | 84.8% | [23] |
| *Physalis peruviana* | peruviose B |  | 78.2% | [23] |
| *Physalis peruviana* | peruviose E |  | 61% | [23] |

Key

DDBT 2-(4-(3,5-dihydroxyphenoxy)-3,5-dihydroxyphenoxy) benzene-1,3,5-triol

# References

1. Dandekar PD, Kotmale AS, Chavan SR, Kadlag PP, Sawant SV, Dhavale DD, RaviKumar A. Insights into the inhibition mechanism of human pancreatic α-amylase, a type 2 diabetes target, by dehydrodieugenol B isolated from *Ocimum tenuiflorum*. ACS Omega. 2021; 6: 1780–1786. https://doi.org/10.1021/acsomega.0c00617

2. Mwakalukwa R, Amen Y, Nagata M, Shimizu K. Postprandial hyperglycemia lowering effect of the isolated compounds from olive mill wastes – an inhibitory activity and kinetics studies on α-glucosidase and α-amylase enzymes. ACS Omega. 2020; 5: 20070–20079. https://doi.org/10.1021/acsomega.0c01622

3. Kawamura-Konishi Y, Watanabe N, Saito M, Nakajima N, Sakaki T, Katayama T, Enomoto T. Isolation of a new phlorotannin, a potent inhibitor of carbohydrate-hydrolyzing enzymes, from the brown alga *Sargassum patens*. J Agric Food Chem. 2012; 60: 5565–5570. https://doi.org/10.1021/jf300165j

4. Sun H, Wang D, Song X, Zhang Y, Ding W, Peng X, Zhang X, Li Y, Ma Y, Wang R, Yu P. Natural prenylchalconaringenins and prenylnaringenins as antidiabetic agents: α-glucosidase and α-amylase inhibition and in vivo antihyperglycemic and antihyperlipidemic effects. J Agric Food Chem. 2017; 65: 1574–1581. https://doi.org/10.1021/acs.jafc.6b05445

5. Ponnusamy S, Haldar S, Mulani F, Zinjarde S, Thulasiram H, RaviKumar A. Gedunin and azadiradione: human pancreatic alpha-amylase inhibiting limonoids from neem (*Azadirachta indica*) as anti-diabetic agents. PLOS ONE. 2015; 10. https://doi.org/10.1371/journal.pone.0140113

6. Ghosh S, More P, Derle A, Patil AB, Markad P, Asok A, Kumbhar N, Shaikh ML, Ramanamurthy B, Shinde VS, Dhavale DD. Diosgenin from *Dioscorea bulbifera*: novel hit for treatment of type II diabetes mellitus with inhibitory activity against α-amylase and α-glucosidase. PLOS ONE. 2014; 9.

https://doi.org/10.1371/journal.pone.0106039

7. Nag G, Das S, Das S, De B. Antioxidant, anti-acetylcholinesterase and anti-glycosidase properties of three species of Swertia, their xanthones and amarogentin: A comparative study. Pharmacogn J. 2015; 7: 117–123. https://doi.org/10.5530/pj.2015.2.6

8. Poongunran J, Perera HK, Jayasinghe L, Fernando IT, Sivakanesan R, Araya H, Fujimoto Y. Bioassay-guided fractionation and identification of α-amylase inhibitors from *Syzygium cumini* leaves. Pharm Biol. 2017; 55: 206–211. https://doi.org/10.1080/13880209.2016.1257031

9. Yonemoto R, Shimada M, Gunawan-Puteri MDPT, Kato E, Kawabata J. α-Amylase inhibitory triterpene from *Abrus precatorius* Leaves, J Agric Food Chem. 2014; 62: 8411–8414. https://doi.org/10.1021/jf502667z

10. Wang H, Du YJ, Song HC. α-Glucosidase and α-amylase inhibitory activities of guava leaves. Food Chem. 2010; 123: 6–13. https://doi.org/10.1016/j.foodchem.2010.03.088

11. Jain V, Viswanatha GL, Manohar D, Shivaprasad HN. Isolation of antidiabetic principle from fruit rinds of *Punica granatum*. Evid Based Complementary Altern Med. 2012. https://doi.org/10.1155/2012/147202

12. Monzon Daza G, Meneses Macias C, Forero AM, Rodríguez J, Aragón M, Jiménez C, Ramos FA, Castellanos L. Identification of α-amylase and α-glucosidase inhibitors and ligularoside A, a new triterpenoid saponin from *Passiflora ligularis* juss (*Sweet granadilla*) leaves, by a nuclear magnetic resonance-based metabolomic study. J Agric Food Chem. 2021; 69: 2919–2931. https://doi.org/10.1021/acs.jafc.0c07850

13. Dehghan H, Salehi P, Amiri MS. Bioassay-guided purification of α-amylase, α-glucosidase inhibitors and DPPH radical scavengers from roots of *Rheum turkestanicum*. Ind Crops Prod. 2018; 117: 303–309. https://doi.org/10.1016/j.indcrop.2018.02.086

14. Ochieng CO, Nyongesa DW, Yamo KO, Onyango JO, Langat MK, Manguro LAO. α-Amylase and α-glucosidase inhibitors from *Zanthoxylum chalybeum* Engl. root bark, Fitoterapia. 2020; 146: 104719. https://doi.org/10.1016/j.fitote.2020.104719

15. Shang C, Gu Y, Koyama T. Major triterpenes, cycloeucalenone and 31-norcyclolaudenone as inhibitors against both α-glucosidase and α-amylase in banana peel. Int J Food Sci Tech. 2021; 56: 3519–3526. https://doi.org/10.1111/ijfs.14978

16. Lulan TYK, Fatmawati S, Santoso M, Ersam T. α-Viniferin as a potential antidiabetic and antiplasmodial extracted from *Dipterocarpus littoralis*. Heliyon. 2020; 6: https://doi.org/10.1016/j.heliyon.2020.e04102

17. Quek A, Kassim NK, Lim PC, Tan DC, Mohammad Latif MA, Ismail A, Shaari K, Awang K. α-Amylase and dipeptidyl peptidase-4 (DPP-4) inhibitory effects of *Melicope latifolia* bark extracts and identification of bioactive constituents using in vitro and in silico approaches. Pharm Biol. 2021; 59: 964–973. https://doi.org/10.1080/13880209.2021.1948065

18. Mbagwu IS, Akah PA, Ajaghaku DL, Ike JC, Okoye FBC. Newboulasides A and B, two new caffeic acid glycosides from *Newbouldia laevis* with α-amylase inhibitory activity. Nat Prod Res. 2020; 36: 1–9. https://doi.org/10.1080/14786419.2020.1799362

19. Cuc NT, Cuong NT, Anh LT, Yen DT, Tai BH, Thu Trang D, Yen PH, Kiem PV, Nam NH, Minh CV, Nhiem NX. Dihydrostilbene glycosides from *Camellia sasanqua* and their α-glucosidase and α-amylase inhibitory activities. Nat Prod Res. 2020; 1–7. https://doi.org/10.1080/14786419.2020.1756802

20. Nickavar B, Abolhasani L. Bioactivity-Guided Separation of an α-Amylase inhibitor flavonoid from *Salvia virgata*. Iran J Pharm Res. 2013; 12: 57–61. https://www.ncbi.nlm.nih.gov/pmc/articles/PMC3813222/

21. Quan NV, Tran HD, Xuan TD, Ahmad A, Dat TD, Khanh TD, Teschke R. Momilactones A and B are α-amylase and α-glucosidase inhibitors. Molecules. 2019; 24: 482. https://www.mdpi.com/1420-3049/24/3/482

22. Centko RM, Ratnaweera PB, Tysoe C, Withers SG, de Silva ED, Andersen RJ. Alpha-glucosidase and alpha-amylase inhibiting thiodiketopiperazines from the endophytic fungus *Setosphaeria rostrata* isolated from the medicinal plant *Costus speciosus* in SriLanka. Phytochem Lett. 2017; 22: 76–80. https://doi.org/10.1016/j.phytol.2017.09.004

23. Bernal CA, Castellanos L, Aragón DM, Martínez-Matamoros D, Jiménez C, Baena Y, Ramos FA. Peruvioses A to F, sucrose esters from the exudate of *Physalis peruviana* fruit as α-amylase inhibitors. Carbohydr Res. 2018; 461: 4–10. https://doi.org/10.1016/j.carres.2018.03.003
