# Supplementary material for: Identification of potential human pancreatic α-amylase inhibitors from natural products by molecular docking, MM/GBSA calculations, MD simulations, and ADMET analysis
Source: PLoS One. 2023 Mar 16;18(3):e0275765. doi: 10.1371/journal.pone.0275765 (PMC10019617; doi:10.1371/journal.pone.0275765)
Supplement: S2 Table — (DOCX) [file pone.0275765.s009.docx]

**Supplementary Material**

**Identification of potential human pancreatic *α*-amylase inhibitors from natural products by molecular docking, MM/GBSA calculations, MD simulations, and ADMET analysis**

Santosh Basnet^1^**^¶^**, Madhav Prasad Ghimire^2&^, Tika Ram Lamichhane^2&^, Rajendra Adhikari^3&^, Achyut Adhikari^1&*^

^1^ Central Department of Chemistry, Tribhuvan University, Kirtipur, Kathmandu, Nepal

^2^ Central Department of Physics, Tribhuvan University, Kirtipur, Kathmandu, Nepal

^3^ Department of Physics, Kathmandu University, Dhulikhel, Nepal

^*^ Corresponding author: [achyutraj05@gmail.com](mailto:achyutraj05@gmail.com)

Table S2. Human pancreatic *α*-amylase

| Name of Target Structure | PDB ID | Resolution (Å) | Reference |
| --- | --- | --- | --- |
| Human Pancreatic *α*-Amylase | 2QV4 | 1.97 | [1] |

# References

1. Maurus R, Begum A, Williams LK, Fredriksen JR, Zhang R, Withers SG, Brayer GD. Alternative catalytic anions differentially modulate human α-amylase activity and specificity. Biochem. 2008; 47: 3332–3344. https://doi.org/10.1021/bi701652t
