# Supplementary material for: Identification of potential human pancreatic α-amylase inhibitors from natural products by molecular docking, MM/GBSA calculations, MD simulations, and ADMET analysis
Source: PLoS One. 2023 Mar 16;18(3):e0275765. doi: 10.1371/journal.pone.0275765 (PMC10019617; doi:10.1371/journal.pone.0275765)
Supplement: S4 Table — (DOCX) [file pone.0275765.s011.docx]

**Supplementary Material**

**Identification of potential human pancreatic *α*-amylase inhibitors from natural products by molecular docking, MM/GBSA calculations, MD simulations, and ADMET analysis**

Santosh Basnet^1^**^¶^**, Madhav Prasad Ghimire^2&^, Tika Ram Lamichhane^2&^, Rajendra Adhikari^3&^, Achyut Adhikari^1&*^

^1^ Central Department of Chemistry, Tribhuvan University, Kirtipur, Kathmandu, Nepal

^2^ Central Department of Physics, Tribhuvan University, Kirtipur, Kathmandu, Nepal

^3^ Department of Physics, Kathmandu University, Dhulikhel, Nepal

^*^ Corresponding author: [achyutraj05@gmail.com](mailto:achyutraj05@gmail.com)

Table S4. Post MM/GBSA (kcal/mol) binding energy of ligands

| SN | Compound | Frame 1 | Frame 500 | Frame 1000 |
| --- | --- | --- | --- | --- |
| 1 | Acarbose | -54.65 | -54.64 | -54.65 |
| 2 | newboulaside B | -77.95 | -77.95 | -77.95 |
| 3 | newboulaside A | -64.39 | -64.39 | -64.39 |
| 4 | quercetin-3-O-*β*-glucoside | -36.44 | -36.44 | -36.44 |
| 5 | sasastilboside A | -65.98 | -65.98 | -65.98 |
