# Supplementary material for: Identification of potential human pancreatic α-amylase inhibitors from natural products by molecular docking, MM/GBSA calculations, MD simulations, and ADMET analysis
Source: PLoS One. 2023 Mar 16;18(3):e0275765. doi: 10.1371/journal.pone.0275765 (PMC10019617; doi:10.1371/journal.pone.0275765)
Supplement: S5 Table — (DOCX) [file pone.0275765.s012.docx]

**Supplementary Material**

**Identification of potential human pancreatic *α*-amylase inhibitors from natural products by molecular docking, MM/GBSA calculations, MD simulations, and ADMET analysis**

Santosh Basnet^1^**^¶^**, Madhav Prasad Ghimire^2&^, Tika Ram Lamichhane^2&^, Rajendra Adhikari^3&^, Achyut Adhikari^1&*^

^1^ Central Department of Chemistry, Tribhuvan University, Kirtipur, Kathmandu, Nepal

^2^ Central Department of Physics, Tribhuvan University, Kirtipur, Kathmandu, Nepal

^3^ Department of Physics, Kathmandu University, Dhulikhel, Nepal

^*^ Corresponding author: [achyutraj05@gmail.com](mailto:achyutraj05@gmail.com)

Table S5. ADMET properties of the acarbose and newboulaside B by QikProp

| Compound | Mol. Wt. | DonorHB | AcceptHB | QPlogPo/w | QPlogS | QPlogKhsa |
| --- | --- | --- | --- | --- | --- | --- |
| Recommended Range | 130.0  –  725.0 | 0.0  –  6.0 | 2.0  –  20.0 | −2.0  –  6.5 | −6.5  –  0.5 | −1.5  –  1.5 |
| acarbose | 645.6 | 14 | 32.1 | -7.254 | 0.739 | -2.601 |
| newboulaside B | 756.7 | 11 | 27.6 | -2.995 | -2.52 | -2.173 |

Key[1]

Donor HB - Number of hydrogen bonds that would be donated

Accept HB - Number of hydrogen bonds that would be accepted

QPlogPo/w - Octanol/water partition coefficient

QPlogS - Aqueous solubility

QPlogkhsa - binding to human serum albumin

# References

1. Jensen BF, Sørensen MD, Kissmeyer AM, Björkling F, Sonne K, Engelsen SB, Nørgaard L. Prediction of in vitro metabolic stability of calcitriol analogs by QSAR. J Comput Aided Mol Des. 2003; 17, 849–859.

https://doi.org/10.1023/B:JCAM.0000021861.31978.da
