# Supplementary material for: Identification of potential human pancreatic α-amylase inhibitors from natural products by molecular docking, MM/GBSA calculations, MD simulations, and ADMET analysis
Source: PLoS One. 2023 Mar 16;18(3):e0275765. doi: 10.1371/journal.pone.0275765 (PMC10019617; doi:10.1371/journal.pone.0275765)
Supplement: S6 Table — (DOCX) [file pone.0275765.s013.docx]

**Supplementary Material**

**Identification of potential human pancreatic *α*-amylase inhibitors from natural products by molecular docking, MM/GBSA calculations, MD simulations, and ADMET analysis**

Santosh Basnet^1^**^¶^**, Madhav Prasad Ghimire^2&^, Tika Ram Lamichhane^2&^, Rajendra Adhikari^3&^, Achyut Adhikari^1&*^

^1^ Central Department of Chemistry, Tribhuvan University, Kirtipur, Kathmandu, Nepal

^2^ Central Department of Physics, Tribhuvan University, Kirtipur, Kathmandu, Nepal

^3^ Department of Physics, Kathmandu University, Dhulikhel, Nepal

^*^ Corresponding author: [achyutraj05@gmail.com](mailto:achyutraj05@gmail.com)

Table S6. ADMET properties of newboulaside B and acarbose by admetSAR

| Property | newboulaside B | | acarbose | |
| --- | --- | --- | --- | --- |
| ADMET Profile | Value | Probability | Value | Probability |
| Human Intestinal Absorption | + | 0.6701 | - | 0.9623 |
| OATP2B1 inhibitor | - | 1 | - | 0.8642 |
| OATP1B1 inhibitor | + | 0.8726 | + | 0.85 |
| OATP1B3 inhibitor | + | 0.9568 | + | 0.9497 |
| MATE1 inhibitor | - | 0.84 | - | 1 |
| OCT2 inhibitor | - | 0.875 | - | 0.95 |
| BSEP inhibitor | + | 0.7961 | - | 0.8825 |
| CYP3A4 inhibition | - | 0.8812 | - | 0.9919 |
| CYP2C9 inhibition | - | 0.8041 | - | 0.8639 |
| CYP2C19 inhibition | - | 0.8473 | - | 0.8109 |
| CYP2D6 inhibition | - | 0.8883 | - | 0.8944 |
| CYP1A2 inhibition | - | 0.8622 | - | 0.852 |
| Carcinogenicity (binary) | - | 0.9571 | - | 0.9857 |
| Ames mutagenesis | - | 0.61 | - | 0.53 |
| Skin sensitization | - | 0.8197 | - | 0.8681 |
| Mitochondrial toxicity | - | 0.525 | + | 0.775 |
| Nephrotoxicity | - | 0.8165 | - | 0.8763 |
| **ADMET profile** | **Value** | **Unit** | **Value** | **Unit** |
| Acute Oral Toxicity | 1.467 | log(1/  (mol/kg)) | 0.944 | log (1/  (mol/kg)) |
