# Supplementary material for: Identification of potential human pancreatic α-amylase inhibitors from natural products by molecular docking, MM/GBSA calculations, MD simulations, and ADMET analysis
Source: PLoS One. 2023 Mar 16;18(3):e0275765. doi: 10.1371/journal.pone.0275765 (PMC10019617; doi:10.1371/journal.pone.0275765)
Supplement: S7 Table — (DOCX) [file pone.0275765.s014.docx]

**Supplementary Material**

**Identification of potential human pancreatic *α*-amylase inhibitors from natural products by molecular docking, MM/GBSA calculations, MD simulations, and ADMET analysis**

Santosh Basnet^1^**^¶^**, Madhav Prasad Ghimire^2&^, Tika Ram Lamichhane^2&^, Rajendra Adhikari^3&^, Achyut Adhikari^1&*^

^1^ Central Department of Chemistry, Tribhuvan University, Kirtipur, Kathmandu, Nepal

^2^ Central Department of Physics, Tribhuvan University, Kirtipur, Kathmandu, Nepal

^3^ Department of Physics, Kathmandu University, Dhulikhel, Nepal

^*^ Corresponding author: [achyutraj05@gmail.com](mailto:achyutraj05@gmail.com)

Table S7. Toxicity profile of newboulaside B and acarbose by ProTox-II

| Property | newboulaside B | | acarbose | |
| --- | --- | --- | --- | --- |
| Toxicity Class | V | | VI | |
| **Target** | **Prediction** | **Probability** | **Prediction** | **Probability** |
| Hepatotoxicity | Inactive | 0.84 | Active | 0.57 |
| Carcinogenicity | Inactive | 0.81 | Inactive | 0.82 |
| Mutagenicity | Inactive | 0.78 | Inactive | 0.72 |
| Cytotoxicity | Inactive | 0.77 | Inactive | 0.68 |
