# Supplementary material for: Identification of potential human pancreatic α-amylase inhibitors from natural products by molecular docking, MM/GBSA calculations, MD simulations, and ADMET analysis
Source: PLoS One. 2023 Mar 16;18(3):e0275765. doi: 10.1371/journal.pone.0275765 (PMC10019617; doi:10.1371/journal.pone.0275765)
Supplement: S8 Table — (DOCX) [file pone.0275765.s015.docx]

**Supplementary Material**

**Identification of potential human pancreatic *α*-amylase inhibitors from natural products by molecular docking, MM/GBSA calculations, MD simulations, and ADMET analysis**

Santosh Basnet^1^**^¶^**, Madhav Prasad Ghimire^2&^, Tika Ram Lamichhane^2&^, Rajendra Adhikari^3&^, Achyut Adhikari^1&*^

^1^ Central Department of Chemistry, Tribhuvan University, Kirtipur, Kathmandu, Nepal

^2^ Central Department of Physics, Tribhuvan University, Kirtipur, Kathmandu, Nepal

^3^ Department of Physics, Kathmandu University, Dhulikhel, Nepal

^*^ Corresponding author: [achyutraj05@gmail.com](mailto:achyutraj05@gmail.com)

Table S8. ADMET properties of newboulaside B (1) and acarbose (2) by pkCSM

| Property | Model Name | **1** | **2** |  | Unit |
| --- | --- | --- | --- | --- | --- |
| Absorption | P-glycoprotein I inhibitor | No | No |  | Categorical (Yes/No) |
| Absorption | P-glycoprotein II inhibitor | No | No |  | Categorical (Yes/No) |
| Distribution | BBB permeability | -2.762 | -2.449 |  | Numeric (log BB) |
| Distribution | CNS permeability | -5.747 | -7.177 |  | Numeric (log PS) |
| Toxicity | hERG I inhibitor | No | No |  | Categorical (Yes/No) |
| Toxicity | hERG II inhibitor | No | Yes |  | Categorical (Yes/No) |
