# Supplementary material for: Identification of potential human pancreatic α-amylase inhibitors from natural products by molecular docking, MM/GBSA calculations, MD simulations, and ADMET analysis
Source: PLoS One. 2023 Mar 16;18(3):e0275765. doi: 10.1371/journal.pone.0275765 (PMC10019617; doi:10.1371/journal.pone.0275765)
Supplement: S9 Table — (DOCX) [file pone.0275765.s016.docx]

**Supplementary Material**

**Identification of potential human pancreatic *α*-amylase inhibitors from natural products by molecular docking, MM/GBSA calculations, MD simulations, and ADMET analysis**

Santosh Basnet^1^**^¶^**, Madhav Prasad Ghimire^2&^, Tika Ram Lamichhane^2&^, Rajendra Adhikari^3&^, Achyut Adhikari^1&*^

^1^ Central Department of Chemistry, Tribhuvan University, Kirtipur, Kathmandu, Nepal

^2^ Central Department of Physics, Tribhuvan University, Kirtipur, Kathmandu, Nepal

^3^ Department of Physics, Kathmandu University, Dhulikhel, Nepal

^*^ Corresponding author: [achyutraj05@gmail.com](mailto:achyutraj05@gmail.com)

Table S9. ADMET properties of newboulaside B and acarbose by SwissADME

| **Property** | | | **newboulaside B** | **acarbose** | | |
| --- | --- | --- | --- | --- | --- | --- |
| **Pharmacokinetics** | | | | | | |
| GI absorption | | Low | | | Low | |
| BBB permeant | | No | | | No | |
| P-gp substrate | | No | | | Yes | |
| Log Kp (skin permeation) | | -12.08 cm/s | | | -16.30 cm/s | |
| **Drug Likeness** | | | | | | |
| Lipinski | No; 3 violations: MW>500, NorO>10, NHorOH>5 | | | | | No; 3 violations: MW>500, NorO>10, NHorOH>5 |
