# Supplementary material for: Identification of potential human pancreatic α-amylase inhibitors from natural products by molecular docking, MM/GBSA calculations, MD simulations, and ADMET analysis
Source: PLoS One. 2023 Mar 16;18(3):e0275765. doi: 10.1371/journal.pone.0275765 (PMC10019617; doi:10.1371/journal.pone.0275765)
Supplement: Attachment — Submitted filename: Response_to_Reviewer.docx [file pone.0275765.s017.docx]

13/01/2023

To

### The Reviewers

# PLOS ONE

Subject: About the Responses to the Comments of Reviewers

# Dear Reviewer,

Herein, we are submitting our revised research manuscript entitled “**Identification of potential human pancreatic *α*-amylase inhibitors from natural products by molecular docking, MM/GBSA calculations, MD simulations, and ADMET analysis**” for publication in your prestigious journal, **PLOS ONE**. We have highlighted where we responded to the reviewer's comments in this revision.

First of all, we would like to express our gratitude for the reviewer's insightful suggestions. In this study, we have proposed Newboulaside B as the potential inhibitor of human pancreatic *α*-amylase. It was isolated in 2020 from *Newbouldia laevis*'s leaves. Previously, researchers had done *in-vitro* on this molecule on fungal *α*-amylase (https://www.tandfonline.com/doi/full/10.1080/14786419.2020.1799362). However, there was a lack of research on human pancreatic *α*-amylase (HPA). Therefore, we short-listed this compound for our research.

**Journal Requirements:**

1. **Please ensure that your manuscript meets PLOS ONE's style requirements, including those for file naming. The PLOS ONE style templates can be found at**

The manuscript is presented in the format specified by the journal.

2. **We note that the grant information you provided in the ‘Funding Information’ and ‘Financial Disclosure’ sections do not match.**

This work was supported by the University Grants Commission, Bhaktapur, Nepal, as part of a thesis grant fellowship award (MRS-77/78-S&T-36).

The funder detail is in the following links. When the proposal was submitted to the UGC Nepal, it was proposed under the title of “In-Silico Evaluation of Different Natural Products as Inhibitors of Alpha-Amylase Enzyme”. UGC provided funding (funding number: MRS-77/78-S&T-36) as dissertation support, but the title has been modified to “Identification of potential human pancreatic α-amylase inhibitors from natural products by molecular docking, MM/GBSA calculations, MD simulations, and ADMET analysis” during journal submission to give more emphasis on finding.

<http://www.ugcnepal.edu.np/uploads/notice/csmNgT.pdf>

3. **Please state what role the funders took in the study.**

The funders had no role in the study design, data collection, analysis, decision to publish, or preparation of the manuscript.

4. **Please remove any funding-related text from the manuscript and let us know how you would like to update your Funding Statement.**

The funding information was not included in the original manuscript and is now only mentioned in the section on the funding statement.

**Comments to the Author**

1. **Is the manuscript technically sound, and do the data support the conclusions?**

Reviewer 1 & Reviewer 2: Partly

We have correlated our computational findings with earlier experimental data in the publication. In the course of the investigation, we also performed a duplicate study to replicate the data.

2. **Has the statistical analysis been performed appropriately and rigorously?**

Reviewer 1:

Thank you for your kind words, reviewer 1. We have used the appropriate tools to analyze the data in accordance with our knowledge and general scientific trends.

Reviewer 2: No comment

3. **Have the authors made all data underlying the findings in their manuscript fully available?**

Reviewer 1: No comment

Reviewer 2: No comment

4. **Is the manuscript presented in an intelligible fashion and written in standard English?**

Reviewer 1: No comment

Reviewer 2: No comment

5. **Review Comments to the Author**

**Reviewer 1:**

We appreciate the reviewer 1's insightful remarks. Your concern has been addressed in the following ways:

1. **Acarbose is a very successful product in clinical application. In order to find new antidiabetic drugs based on α-amylase, it is necessary to consider the existing problems of acarbose in the design of the manuscript. The results of the present comparison with acarbose are not particularly significant.**

Thank you for your valuable remarks. Acarbose is one of the effective candidates to control type 2 diabetes, reviewer 1 is correct. We also utilized it as the positive control because of this (reference compound ). Despite the candidate's success, type 2 diabetes patients continue to be an issue. They are not entirely recovering. Acarbose also shows certain adverse effects in the abdomen. Therefore our hypothesis was to search for an alternative candidate. To some extent, we tried to address it in the manuscript by adding reference 10 (references 6-10). In our opinion, this literature will be sufficient, which will also help to make the manuscript concise.

2. **Acarbose directly inhibits α-glycosidase in the intestine, and this paper is to find an inhibitor of α-amylase. Although these two enzymes have similarities, it is a question to take acarbose as a positive control. It is recommended to use the compounds with the inhibitory effect of α-amylase confirmed in the literature as a positive control. Acarbose as the positive control**

We appreciate your kind words. Yes, acarbose is used as a positive control against α-glucosidase. According to the literature (taken co-crystalized target (2QV4) and supplementary references (Table S1 and Table S2)), it also inhibits α-amylase. We don't think it needs to be written in the manuscript because doing so would make it less concise.

3. **Without entering the blood, the drug can directly inhibit α-amylase or α-glycosidase in the intestine to play a hypoglycemic effect. The ideal result is that the drug does not be absorbed into the blood. The ADMET result in the paper is not suitable.**

Thank you for the insightful feedback. Yes, the inhibitor's role is to inhibit α-amylase. However, we cannot assert that the body does not absorb the substances we ingest. We cannot directly state that our body's efflux inhibitor appears in the form of faeces. Due to this, we also included ADMET analysis in our research.

4. **According to the MM/GBSA results, only newboulaside B had a binding free energy similar to acarbose. The other three components ( newboulaside A, quercetin-3-O-β-glucoside, and sasastilboside A) have good docking scores but not free energies. It is suggested to use MD results to calculate the binding free energy and verify the binding ability of these components with α-amylase**.

We appreciate your insightful comment regarding the post-MM/GBSA binding energy. Using implicit solvent (VSGB), we have computed binding energy at frames 1, 500, and 1000. Due to orientation change after simulation, we observed binding energies greater than pre-MM/GBSA binding energies. From this, we also found neboulaside B as potential candidate. Updated information for all frames is highlighted in the manuscript's sections 2.5 and 3.4 as well as the supplementary material (Table S4). The number of the supplementary table has also been changed as a result of the inclusion of the post-MM/GBSA data (Table S4).

**Reviewer 2:**

**The authors have carried out extensive in silico studies on the interactions of newboulaside B as a human pancreatic amylase (HPA) inhibitor. The computational study has been well designed and analysed. After target protein (HPA) and newboulaside B preparation and energy minimization, molecular docking was carried out and MM/GBSA binding energies calculated using GLIDE. Further MD simulations taking into consideration the solvation of Na+ and Cl- ions it was ascertained that newboulaside B interaction with HPA was fairly stable with minimal deviation and interacted with the active site residues. Further the ADMET studies have also been carried out to check for its toxicity .
However, it is to be noted that newboulaside B inhibited fungal amylase with an IC 50 of 4.44 µg/mL ( ref no. 28). No information on its inhibition or kinetics is available on human pancreatic amylase (HPA) . To validate the in silico data it is necessary to carry out the enzyme inhibition and its kinetics to prove it as a completive inhibitor which the authors are stating. It has been very often seen that the mode of inhibition (competive, uncompetietive , non-competietive) in experimental studies differs from computational data. Hence while computational studies suggest good leads and probable modes of binding, it it relevant to validate their data experimentally.**

For your insightful criticism of the paper, reviewer 2 is gratefully acknowledged.

Previously, newboulaside B’s experiment was done with fungal α-amylase with IC_50_ value (4.44 µg/mL). We encountered a challenge when trying to conduct research with these compounds and human pancreatic α-amylase. The compound's source, which was recently determined and is also stated in the document, is Africa. Geographical constraints made it difficult for us to conduct the experiment at this time. Therefore, we tried to address such concerns by correlating them with previous experiments.

To address correlation concern, we also did a simulation study with fungal α-amylase (7TAA), and we also correlated the active site region of human pancreatic α-amylase and fungal α-amylase. Active residues are nearly aligned, as evidenced by structural similarities, with the exception of the loop. The addition of references clarifies them as well (manuscript references: 37 and 38).  Newboulaside B is more stable with HPA than fungal α-amylase, according to the simulation results, which are updated in the text (section 3.4) and the supplemental material (Fig. S6 and Fig. S7) as well. These findings support the HPA-inhibiting properties of newboulaside B.

In addition to the revision described above, the abstract, conclusions, and supporting information have all undergone minor revisions, which are mostly highlighted in the text.

Based on the above-mentioned new finding, we think our work is suitable for publication in your esteemed journal. we thus request your kind consideration and necessary action.

I hereby declare that all the authors involved in this work have approval for the submission process.

Achyut Adhikari, PhD

Associate Professor,

Central Department of Chemistry, Tribhuvan University,

Kirtipur-44613, Kathmandu, Nepal

**Email**: [achyutraj05@gmail.com](mailto:achyutraj05@gmail.com); **Mobile Number**: +977-9861490245
