# Supplementary material for: Identification of potential human pancreatic α-amylase inhibitors from natural products by molecular docking, MM/GBSA calculations, MD simulations, and ADMET analysis
Source: PLoS One. 2023 Mar 16;18(3):e0275765. doi: 10.1371/journal.pone.0275765 (PMC10019617; doi:10.1371/journal.pone.0275765)
Supplement: Attachment — Submitted filename: Response_to_Reviewer.docx [file pone.0275765.s018.docx]

20/02/2023

To

### The Reviewer

# PLOS ONE

Subject: About the Responses to the Comments of Reviewers (Minor Revision)

# Dear Reviewer,

First of all, we would like to express our gratitude for the reviewer's insightful suggestions and kind words. Herein, we are submitting our revised research manuscript entitled “**Identification of potential human pancreatic *α*-amylase inhibitors from natural products by molecular docking, MM/GBSA calculations, MD simulations, and ADMET analysis**” for publication in your prestigious journal, **PLOS ONE**. We have responded to the reviewers' comments with track changes.

In this study, we have proposed Newboulaside B as the potential inhibitor of human pancreatic *α*-amylase. It was isolated in 2020 from *Newbouldia laevis*'s leaves. Previously, researchers had done *in-vitro* on this molecule on fungal *α*-amylase (https://www.tandfonline.com/doi/full/10.1080/14786419.2020.1799362). However, there was a lack of research on human pancreatic *α*-amylase (HPA). Therefore, we also short-listed this compound for our research. Our study also correlates the candidate with the previous experiment. Therefore, in the future, we hope that this molecule will be useful in the pharmaceutical industry as a lead/optimized molecule for drug discovery.

**Comments to the Author**

1. **If the authors have adequately addressed your comments raised in a previous round of review and you feel that this manuscript is now acceptable for publication, you may indicate that here to bypass the “Comments to the Author” section, enter your conflict of interest statement in the “Confidential to Editor” section, and submit your "Accept" recommendation.**

Reviewer #1: (No Response)

Reviewer #2: All comments have been addressed

2. **Is the manuscript technically sound, and do the data support the conclusions?**

Reviewer #1: Partly

Reviewer #2: Yes

3. **Has the statistical analysis been performed appropriately and rigorously?**

Reviewer #1: Yes

Reviewer #2: Yes

4. **Have the authors made all data underlying the findings in their manuscript fully available?**

Reviewer #1: Yes

Reviewer #2: Yes

5. **Is the manuscript presented in an intelligible fashion and written in standard English?**

Reviewer #1: (No Response)

Reviewer #2: Yes

6. **Review Comments to the Author**

**Reviewer 1:**

We appreciate reviewer 1's insightful remarks. Your concern has been addressed in the following ways:

1. **Desmond has a FEP module, which can calculate the free energy according to the MD trajectory. Is it appropriate to use the Post MM/GBSA binding energy method? It is suggested to provide the literature for using a similar calculation method.**

Thank you for your valuable remarks regarding the FEP module of Desmond for the post-analysis. Yes, we agree with your comment’s intention. We must say that for the post-analysis, despite being expensive, the FEP of Schrodinger is the better technique than MM/GBSA approach. Unfortunately, we must say that recently our license date of Schrodinger has expired, so we became unable to calculate FEP from Desmond. Therefore, we have some compulsion to rely on the previously followed approach [i.e. MM/GBSA approach]. Yes, MM/GBSA is also an appropriate tool for the post-binding energy analysis, which has been clarified in the manuscript by adding references [24,25,26] in section 2.5 (post-MM/GBSA binding energy calculation).

2. **The top structure found in this manuscript has been reported in literature experiments with similar activity. If the interaction mode between ligand and target cannot be deeply analyzed to provide direction for the discovery of new structures, the value of the manuscript will be low. Please discuss this in the manuscript together with appropriate references.**

Thank you for your insightful remarks. Yes, we have analyzed our top molecule (newboulaside B) interaction deeply. In the manuscript’s molecular docking section 3.2 (paragraphs 1, 2, 3 with reference 32), we have analyzed molecular interaction.

We all know that molecular docking is not an accurate and sufficient tool to analyze binding interaction. In the actual environment, molecules do not behave rigidly as in docking, they behave flexibly. Therefore, they may interact differently. Thus, these interactions were further analyzed deeply in the molecular dynamics section 3.3 (paragraphs 4, 5, 8) in every frame of the simulation by using the stacked bar, timeline chart, and the interaction percentage time. Therefore, the interaction of protein and ligand, by correlating, has been analyzed in both rigid and flexible forms profoundly.

**Reviewer 2:**

**The authors have addressed the queries in the revised manuscript. The authors have identified 4 potential HPA inhibitors using an in silico approach. Further ADMET analysis on the molecules has also been performed. The work has been carried out thoroughly though validation of the molecules by experimental methods would have added value to the paper.**

Based on the above-mentioned new finding, we think our work is suitable for publication in your esteemed journal. we thus request your kind consideration and necessary action.

I hereby declare that all the authors involved in this work have approval for the submission process.

Achyut Adhikari, PhD

Associate Professor,

Central Department of Chemistry, Tribhuvan University,

Kirtipur-44613, Kathmandu, Nepal

**Email**: [achyutraj05@gmail.com](mailto:achyutraj05@gmail.com); **Mobile Number**: +977-9861490245
